# From mummies to internal mammaries: a historical perspective on the discovery of atherosclerosis

**DOI:** 10.1530/VB-25-0013

**Published:** 2026-07-16

**Authors:** Vincent Q Sier, Ferdi Akca, Paul T Sergeant, Jan M De Raet, Andrew J Nelson, Paul H A Quax, Niels Verberkmoes, Alan Dardik, Margreet R de Vries

**Affiliations:** ^1^ Department of Surgery, Leiden University Medical Center, Leiden, The Netherlands; ^2^ Einthoven Laboratory for Experimental Vascular Medicine, Leiden University Medical Center, Leiden, The Netherlands; ^3^ Department of Cardiothoracic Surgery, Catharina Hospital, Eindhoven, The Netherlands; ^4^ Department of Cardiac Surgery, KU Leuven, Leuven, Belgium; ^5^ Department of Cardiac Surgery, Saint-Jean Hospital, Brussels, Belgium; ^6^ Department of Anthropology, University of Western Ontario, London, Ontario, Canada; ^7^ Department of Surgery, Icahn School of Medicine at Mount Sinai, New York, New York, USA; ^8^ Department of Cardiology, Brigham & Women’s Hospital and Harvard Medical School, Boston, Massachusetts, USA

**Keywords:** atherosclerosis, vascular disease, history, arterial occlusive disease, cardiovascular

## Abstract

The aim of this study was to explore the historical evolution of atherosclerosis, from early understanding of the function of the heart and blood vessels to the discovery of arterial occlusive disease. This narrative review draws on historical medical texts, archaeological findings, and recent literature. It synthesizes developments in cardiovascular understanding across ancient, classical, and modern eras, with attention to changing paradigms. Evidence of atherosclerosis has been observed in mummified remains from ancient civilizations, predating any formal understanding of the cardiovascular system. In classical antiquity, the heart and vessels were often assigned symbolic or spiritual meaning, gradually giving way to empirical inquiry and anatomical study. Eventually, atherosclerosis came to be recognized as a multifactorial disease with significant clinical consequences. The understanding of atherosclerosis has evolved through centuries of speculation, anatomical discovery, and technological innovation. These perspectives provide a non-exhaustive overview of historical concepts of the cardiovascular system and atherosclerosis in particular.

## Uncovering the mysteries of the heart and its blood vessels

**Figure 1:**
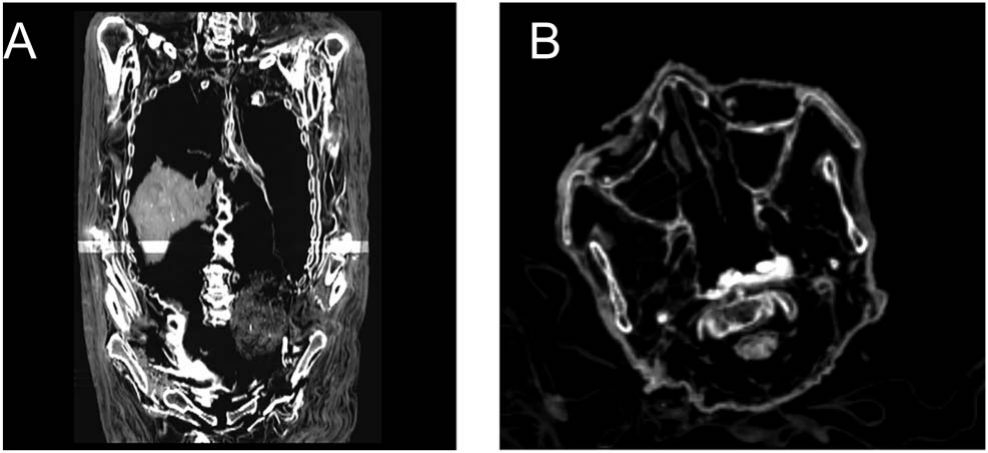
(A) Three-dimensional rendering of the thoracic cavity of the Mummy of Harerem (Leiden University, National Museum of Antiquities, Cat. 11) (44). In addition to filling material, as clearly visible in the right thoracic cavity (sand or mud), structures pertaining to the pleura, heart, and great vessels can be observed on the left side (44). (B) Lady Hudson is a Roman Period Mummy (30 BC to 395 AD) and thought to be from a wealthy family during her life. Examination has identified atherosclerosis in many of her blood vessels, including the carotid arteries (45). CT scans of these mummies were obtained through and are courtesy of the IMPACT database (46).

The discovery of atherosclerosis as a multi-factorial chronic disease of the arteries is tightly linked to its clinical manifestations in the form of cardiac and limb ischemia. Evidence shows that atherosclerotic calcifications were already prevalent in preindustrial populations (1, 2). Computed tomography (CT) images of over 200 mummified humans from various cultures, including ancient Egyptians, Peruvians, the Ancestral Puebloans of southwest America, Andean highland Bolivians, the Unangan of the Aleutian Islands, 16th-century Greenlandic Inuit, Gobi Desert pastoralists, and 19th-century African Americans, demonstrate the presence of calcified blood vessels, including the aorta, carotid, iliac, and coronary arteries (Fig. 1).

### The cardiovascular system as the center of the human spirit and bodily processes

Despite the evidence of calcified atherosclerotic lesions in very early human civilizations (3) (notable work on arterial lesions in mummies was conducted by paleopathologist Sir Marc Armand Ruffer (1859–1917 AD) in the early 20th century; through dissection, he identified atherosclerosis with calcified plaques in various arteries of embalmed mummies (1580 BC–525 AD)), a true conceptual understanding of the disease required significant advancements in anatomical and physiological knowledge of the cardiovascular system. As early as 3500 BC in ancient Egypt, the heart was already considered the center of a complex network of vessels. The Egyptians believed that it was necessary to transport substances such as air, saliva, urine, and diseases throughout the human body (4, 5). They related their own palpable pulse to the organ of the heart. There is even evidence that Egyptian civilization was advanced enough to be able to recognize and describe certain clinical symptoms of cardiac ischemia (5).

Building upon the early insights of the Egyptians into the heart and blood vessels, ancient Greece became the intellectual epicenter of the ancient world. During the Homeric period (∼1100–750 BC), scholars approached the heart with a symbolic and religious worldview (6). In Homer’s Iliad, the heart (kardia) was seen not merely as an organ but as the seat of courage, strength, and thumos, an ‘inner fire’ essential to a warrior’s will (7, 8).

From the 5th century BC onward, the Hippocratic school marked a pivotal shift from mystical interpretations of the heart to a more systematic and empirical approach. Texts from this period described the heart as the central organ sustaining life. They also introduced the concept that the lungs served to cool the heart, which was believed to generate excessive heat due to its continuous activity (4, 9). Aristotle (384–322 BC) further refined this idea and described the heart as the spiritual and reflective center of the body. Consisting of three cavities, likely viewing the right atrium as the distal venous dilation of the caval vein, the heart was in his view central to life and connected to a system of arteries and veins (10, 11).

Significant anatomical advancements emerged from the anatomists of the Alexandrian school (12, 13). While Herophilus of Chalcedon (335–280 BC) observed that arteries had thicker, more elastic walls compared with veins and linked their pulsation to the movement of blood, it was Erasistratus of Ceos (304–250 BC) who proposed their distinct functions within the circulatory system. He proposed that arteries carried pneuma (vital air or spirit), while veins transported blood.

The Greek Galen of Pergamum (c. 129–216 AD), serving as the personal physician to emperor Marcus Aurelius (121–180 AD), synthesized and experimented with the prevailing concepts in the first centuries AD, creating a model of the cardiovascular system that would dominate medical understanding for well over a thousand years. Galen described arteries and veins to belong to different systems: he believed venous blood to originate from the liver, while arterial blood carrying ‘vital pneuma’ flowed through the arteries (14). After returning to the heart, venous blood passed through tiny pores in the interventricular septum to reach the left system, where it was vitalized before its distribution throughout the body. Etymologically, it is fitting that the word artery (Latin: artēria) originates from ‘windpipe’, while vein (Latin: vēna) traces its roots to ‘watercourse’.

**Table:** 

**Historical framework: Eastern perspectives on the heart and blood vessels** ❖ Beyond the traditional Egyptian, Greek, and Roman literature, historical records from other regions of the world provide valuable insights into the global understanding of the cardiovascular system in the centuries leading up to and from the start of the first millennium. ❖ In ancient India (circa 600 BC), texts such as *Sushruta Samhita* and *Charaka Samhita* described the heart as the seat of consciousness and the source of *prana* (life energy). The heart transmitted energy through *nadis* (channels), later identified as veins (*siras*), arteries (*dhamanis*), and flows (*srotas*). The liver, rather than the lungs, was seen as central to blood purification and circulation (15). ❖ The Sassanid Empire, centered in ancient Persia (modern-day Iran, 224–637 AD), viewed the heart as the source of vitality and linked blood to nourishment and disease. They compared vessels to rivers, proposed early ideas of pulmonary circulation, and associated blood impurities (invisible monsters) with infection (16). ❖ In ancient Chinese medicine, Mai (vessels) were described during the Warring States Period (476–221 BC) in texts such as the *Cauterization Canon of the Eleven Vessels* (17). These vessels were believed to connect internal organs to the body’s extremities and external environment, facilitating the flow of *Qi* (vital energy) rather than blood. The heart’s role in circulation was minimal, while the liver was considered central (18).

### Breaking with anatomical dogmata

Intellectuals in the Islamic Golden Age, such as Ibn al-Nafis (1213–1288 AD), started to reject some of Galen’s anatomical contentions. Notably, instead of interventricular pores, he correctly hypothesized that blood moved from the right ventricle to the left via the lungs, a fundamental insight into pulmonary circulation.

It was not until the Renaissance that a turning point in anatomical understanding began to emerge in Europe, as scholars would also start to challenge the long-held views of the Galen School. Leonardo da Vinci (1452–1519 AD) was among the first to meticulously map the anatomy of the cardiovascular system. Building upon this early progress, the Dutch physician and anatomist Andreas Vesalius (1514–1564 AD) questioned the anatomic descriptions of human organs by Galen, given the fact that a fair share of his work was based on dissection of animals. Vesalius’ critique of the Galenic dogma was presented in his De Humani Corporis Fabrica Libri Septem (On the Fabric of the Human Body in Seven Books) (19). Interestingly, Vesalius did not present an alternative to the prevalent physiological theories, whereas his student Realdo Colombo (1516–1559 AD) described the passage of blood from the right ventricle to the lungs and back to the left ventricle via the pulmonary vessels (20). Similarly, theologian and physician Michael Servetus (1511–1553 AD) was the first to reject the concept of interventricular pores in the western world and described pulmonary transit of blood in his work Christianismi Restitutio (21).

It was English physician William Harvey (1578–1657 AD) who truly transformed the understanding of the cardiovascular system at the time. In his Exercitatio Anatomica de Motu Cordis et Sanguinis in Animalibus (An Anatomical Study on the Motion of the Heart and Blood in Animals), Harvey demonstrated that blood circulates in a continuous loop, propelled by the heart as a pump through a closed system of arteries and veins (22). In 1661, Marcello Malpighi (1629–1694 AD) discovered the existence of capillaries in frog lungs by making use of the recently invented compound microscope (23, 24). This discovery completed the anatomical model of the circulatory system that Harvey had postulated.

### Vienna as a hub in anatomical science: achievements and ethical questions

**Figure 2:**
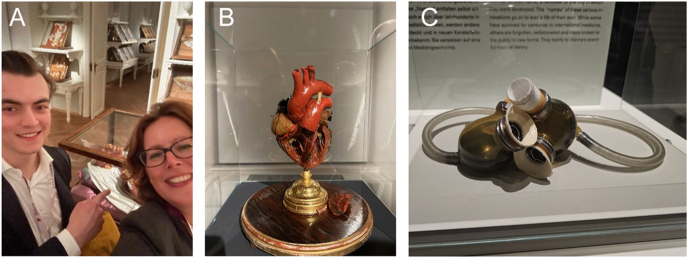
(A) Two researchers exploring the wax model collection of Emperors Leopold II and Joseph II at the Josephinum medical history museum. The Josephinum opened its doors in 1785 as the central hub of the newly established military surgical-medical academy in Vienna, Austria. (B) Anatomical wax model of the human heart with exposed internal structures of the left and right ventricles. (C) The ‘New Vienna Heart’, a pneumatically powered artificial heart first implanted in a patient in 1989. The development of the artificial heart was catalyzed by earlier work of Willem Kolff (1911–2009 AD), who, besides performing the first successful dialysis procedure (1945), laid the conceptual and technical groundwork for cardiopulmonary bypass from the 1930s onward. He was involved in the development of the first artificial heart to be successfully implanted in a human (1982, Jarvik-7, patient: Barney Clark). The models and device shown in this figure are part of the collections and exhibitions of the Medical University of Vienna.

Emperor Joseph II of Vienna (1741–1790 AD) was key in turning the University of Vienna into a major center for anatomical research. He aimed to improve medical education and ordered 1,192 wax anatomical models, inspired by Florence’s La Specola. Together with detailed diagrams and multilingual explanations, these models formed one of the best teaching collections of the time (Fig. 2), attracting scholars and doctors from across Europe to Vienna.

By the early 20th century, Vienna was seen as the world leader in anatomical research. In this setting, Eduard Pernkopf (1888–1955 AD) created his Topographische Anatomie des Menschen, published between 1937 and 1957. Pernkopf’s atlas became the gold standard in anatomical illustration, known for its incredible detail, especially of vascular and neurovascular structures. Surgeons worldwide used the atlas to guide complex procedures such as coronary artery bypass grafting (CABG). The atlas’s scientific value as the gold standard of anatomy knowledge, however, is overshadowed by its unethical origins. Produced under the Nazi regime, this material is now considered to have been unethically gathered (25).

With anatomy better understood, medical focus began to shift toward the diseases affecting these structures. This transition paved the way for key discoveries in cardiovascular pathology, including the early understanding of arterial occlusive disease, which would soon become a major focus for clinicians and researchers alike.

### Discovery of the building bricks of arterial occlusive disease

Following the foundational anatomical mapping of cardiovascular structures during the Renaissance, pathologists and clinicians in the following centuries shifted their attention toward disease affecting the circulatory system. William Heberden (1710–1801 AD) documented a ‘disorder of the breast’ he termed angina pectoris, characterized by tightness of the chest, anxiety, and pain radiating to the left arm in 1772 (26). Approximately 50 years later, the inventor of the stethoscope René Laennec (1781–1826 AD) described instances where veins and arteries were completely obstructed by fibrin, which adhered to the vessel walls and narrowed their caliber (27, 28). Surgeon John Hunter (1728–1793 AD) is recognized to have demonstrated the principle of collateral circulation through a well-known anecdote crediting this insight to experiments on a stag. Purportedly, ligation of the carotid artery stimulated alternative blood flow to sustain the antler’s growth, although this story has been critiqued for lacking direct evidence (29, 30). Veterinarian-surgeon Jean-François Bouley (1787–1855 AD) documented the first case of intermittent claudication in a horse several years later. He attributed lameness in their leg to obstructing clots in the femoral arteries (31, 32, 33, 34).

Around the same period, surgeon-pathologist Jean Lobstein (1777–1835 AD) first introduced the term ‘arteriosclerosis’, deriving it from the Greek words ‘arteria’ (artery) and ‘sclerosis’ (hardening), to describe the stiffening and thickening he observed in arterial walls (35). Lobstein noticed that arteriosclerosis affected larger arterial trunks, especially in the lower extremities, which were prone to ossification compared with arteries in the upper body. He noted that calcifications frequently occurred in curved sections, such as parts of the carotid and splenic artery. Morphologically, the affected arteries displayed a pale yellow to reddish-brown coloration, with thickened, dilated walls.

Building on Lobstein’s early observations, physician-pathologist Rudolf Virchow (1821–1902 AD) examined plaques under the microscope and observed inflammatory cells in the vascular wall (36). Together with insights of others such as Joseph Hodgson (1788–1869 AD) and Sir William Osler (1849–1919 AD), it became apparent that pathological arterial occlusion was not merely caused by a passive thickening of the vascular walls but was an active, inflammatory condition (37, 38, 39). Despite these early observations, the inflammatory component of arterial disease was largely overlooked until the late 20th century, when pathologist Russell Ross (1929–1999 AD) established the response-to-injury model (40). This seemingly delayed recognition was, at least in part, due to the shift in vascular biologists’ focus toward a newly identified ‘fatty’ component of vessel walls.

### Birth of atherosclerosis as a multifactorial disease

The term ‘atherosclerosis’ (from ‘athero’; gruel or paste) began to take shape in the early 20th century as scientists increasingly recognized a distinct form of arteriosclerosis characterized by lipid-rich plaques within the arterial walls. Notably, Russian pathologist Nikolaj Nikolajewitsch Anitschkow (1885–1964 AD) demonstrated that feeding rabbits a cholesterol-rich diet induced plaque buildup, highlighting the role of lipids in this specific form of arterial disease (41, 42). Clinically, it was also around this time that James B. Herrick (1861–1954 AD), an American physician, published a definitive paper describing how clinical symptoms of myocardial infarction could be caused by obstruction of arterial flow (43).

## Conclusion

In summary, atherosclerosis has been present throughout human history, but its true discovery depended on a foundational understanding of the heart and blood vessels. As knowledge of the cardiovascular system advanced, atherosclerosis eventually emerged as a distinct disease entity. Today, researchers continue to explore its multifactorial nature, with new medications and treatment options on the horizon. Alongside these developments, surgical treatment strategies have also played a key role, although that particular history warrants a tale of its own.

## Declaration of interest

All authors declare no conflicts of interest. PHA Quax serves as a co-Editor-in-Chief of *Vascular Biology*. He was not involved in the review or editorial process for this article, on which he is listed as an author.

## Funding

This work was supported by a Leiden University Medical Center MD/PhD research grant to VQS.
